# ICON: chronic rhinosinusitis

**DOI:** 10.1186/1939-4551-7-25

**Published:** 2014-10-27

**Authors:** Claus Bachert, Ruby Pawankar, Luo Zhang, Chaweewan Bunnag, Wytske J Fokkens, Daniel L Hamilos, Orathai Jirapongsananuruk, Robert Kern, Eli O Meltzer, Joaquim Mullol, Robert Naclerio, Renata Pilan, Chae-Seo Rhee, Harumi Suzaki, Richard Voegels, Michael Blaiss

**Affiliations:** 1 Upper Airways Research Laboratory (URL), University Hospital Ghent, Belgium; 2 Div. of Allergy, Dept. of Pediatrics, Nippon Medical School, Tokyo, Japan; 3 Beijing Key Laboratory of Nasal diseases, Beijing Institute of Otolaryngology, Beijing, China; 4 Faculty of Medicine, Siriraj Hospital Mahidol University, Bangkok, Thailand; 5 Department of Otorhinolaryngology, Academic Medical Centre, Amsterdam, The Netherlands; 6 Division of Rheumatology, Allergy & Immunology, Massachusetts General Hospital, Boston, MA USA; 7 Department of Otolaryngology Head and Neck Surgery, Northwestern University Feinberg School of Medicine, Chicago, Illinois USA; 8 Allergy & Asthma Medical Group & Research Center, San Diego, California USA; 9 Rhinology Unit & Smell Clinic, Hospital Clínic – IDIBAPS, Barcelona, Catalonia Spain; 10 Section of Otolaryngology Head and Neck Surgery, Department of Surgery, University of Chicago, Chicago, Illinois USA; 11 Department of Otorhinolaryngology, Clinics Hospital/University of Sao Paulo Medical School, Brazil; 12 Department of Otorhinolaryngology-Head and Neck Surgery, Seoul National University Bundang Hospital, Seongnam, Seoul National University College of Medicine, Seoul, Korea; 13 Dept. of Otorhinolaryngology, Showa University, Tokyo, Japan; 14 Department of Rhinology, University of Sao Paulo Medical School, Sao Paulo, Brazil; 15 University of Tennessee Health Science Center, Memphis, Tennessee USA

**Keywords:** Chronic rhinosinusitis, Pharmacoeconomics, Pathophysiology, Phenotypes, Genetics, Co-morbidities, Treatment, Biologicals, Unmet needs

## Abstract

Chronic rhinosinusitis (CRS) is a public health problem that has a significant socio-economic impact. Moreover, the complexity of this disease due to its heterogeneous nature based on the underlying pathophysiology - leading to different disease variants - further complicates our understanding and directions for the most appropriate targeted treatment strategies. Several International/national guidelines/position papers and/or consensus documents are available that present the current knowledge and treatment strategies for CRS. Yet there are many challenges to the management of CRS especially in the case of the more severe and refractory forms of disease. Therefore, the International Collaboration in Asthma, Allergy and Immunology (iCAALL), a collaboration between EAACI, AAAAI, ACAAI, and WAO, has decided to propose an International Consensus (ICON) on Chronic Rhinosinusitis. The purpose of this ICON on CRS is to highlight the key common messages from the existing guidelines, the differences in recommendations as well as the gaps in our current knowledge of CRS, thus providing a concise reference. In this document we discuss the definition of the disease, its relevance, pharmacoeconomics, pathophysiology, phenotypes and endotypes, genetics and risk factors, natural history and co-morbidities as well as clinical manifestations and treatment options in both adults and children comprising pharmacotherapy, surgical interventions and more recent biological approaches. Finally, we have also highlighted the unmet needs that wait to be addressed through future research.

**Electronic supplementary material:**

The online version of this article (doi:10.1186/1939-4551-7-25) contains supplementary material, which is available to authorized users.

## Definition of disease

Rhinosinusitis (RS) is a significant health problem which seems to mirror the increasing frequency of allergic rhinitis and which results in a large financial burden on society. Rhinosinusitis is a broad umbrella term covering multiple disease entities, including acute RS (ARS), CRS with nasal polyps (CRSwNP) and CRS without nasal polyps (CRSsNP). In this document only Chronic Rhinosinusitis with or without polyps will be discussed. The last decade has seen the development of a number of guidelines, consensus documents and position papers on the epidemiology, diagnosis and treatment of RS [1–6]. All guidelines being published in recent years have adopted the term r’hinosinusitis’ instead of sinusitis. Recent data have demonstrated that CRS affects approximately 5–15% of the general population [7–11]. The prevalence of doctor-diagnosed CRS was found to be 2-4% [9].

Chronic Rhinosinusitis in adults is in most guidelines defined as an inflammation of the nose and the paranasal sinuses characterised by at least 8-12 weeks of at least 2 symptoms, like nasal blockage/obstruction/congestion, nasal discharge (anterior/posterior nasal drip), facial pain/pressure and/or reduction or loss of smell and either endoscopic signs of disease or relevant CT scan changes. Confirmation of sinus disease using an objective measure is required because the symptoms can be nonspecific and mimicked by several disease entities (eg, upper respiratory tract infection, (allergic) rhinitis, migraine). Conversely, in the absence of symptoms, diagnosis of CRS based on radiology alone is not appropriate because of a high incidence of radiological anomalies on CT scans in normal individuals. Thus, the presence of symptoms plus an objective finding are necessary [4]. Addition of nasal endoscopy to symptom assessment substantially increased diagnostic accuracy in confirming the presence of CRS using sinus CT as the criterion standard [12]. CRS is usually further categorized based on the absence or presence of nasal polyps (CRS without nasal polyps, CRSsNP; or CRS with nasal polyps, CRSwNP). Although both are characterized by mucopurulent drainage and nasal obstruction, CRSsNP is frequently associated with facial pain/pressure/fullness whereas CRSwNP is frequently characterized by hyposmia. Nasal polyps (CRSwNP) are defined as bilateral pedunculated lesions as opposed to cobblestoned mucosa, endoscopically visualised in middle meatus [5]. In some guidelines further categorisation or subanalysis of patient groups with CRSwNP is advised e.g. into allergic fungal rhinosinusitis [1], aspirin exacerbated respiratory disease [1–4], and/or cystic fibrosis [1, 5, 6, 13].

The definition in children is less well developed. In the EPOS2012 guidelines, chronic rhinosinusitis in children is defined as an inflammation of the nose and the paranasal sinuses characterised by at least 8-12 weeks of at least symptoms of nasal blockage/obstruction/congestion and/or nasal discharge (anterior/posterior nasal drip), combined with facial pain/pressure and/or reduction of smell or cough and either endoscopic signs of disease or relevant CT scan changes.

The goal of CRS treatment is to achieve and maintain clinical control and avoid complications. The terms control of disease and difficult-to-treat rhinosinusitis have been defined. Control is defined as a disease state in which the patients do not have symptoms or the symptoms are not bothersome, if possible combined with a healthy or almost healthy mucosa and only the need for local medication. Difficult-to-treat rhinosinusitis was defined as persistent symptoms of rhinosinusitis despite appropriate treatment (recommended medication and surgery). A significant, although presently unknown, percentage of patients with CRS continue to experience bothersome symptoms despite adequate treatment. This group with so-called severe chronic upper airway disease (SCUAD) represents a therapeutic challenge [14].

## Prevalence of chronic rhinosinusitis

Studies designed to investigate Chronic Rhinosinusitis (CRS) epidemiology play an important role in assessing its distribution, analyzing risk factors, and promoting public health policies. Epidemiological data on rhinosinusitis are scarce, and study methods and response rates vary widely.

**Figure 1:**
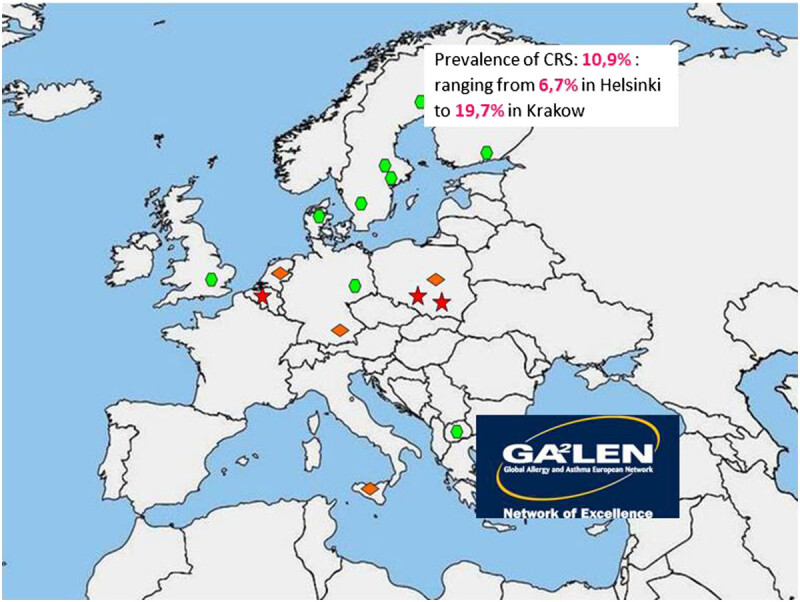
**Prevalence of CRS.** The GA2LEN study. Reported prevalence of CRS throughout Europe [9]).

As suggested by the European Position Paper on Rhinosinusitis and Nasal Polyps (EPOS 2007 and 2012), from an epidemiological standpoint, chronic rhinosinusitis (with or without nasal polyps) in adults is defined as: presence of two or more symptoms one of which should be either nasal blockage/obstruction/congestion or nasal discharge (anterior/posterior nasal drip): ± facial pain/pressure; ± reduction/loss of smell; and symptoms must be present for more than 12 weeks [5, 13]. In a recent multicenter study performed as part of the Global Allergy and Asthma European Network project (GA2LEN), a postal questionnaire with the EPOS criteria was sent to a random sample of adults aged 15-75 years in 19 centers of 12 countries in Europe. The GA2LEN study concluded that the overall prevalence of CRS by EP3OS criteria was 10.9% (range 6.9- 27.1) [9] (Figure 1) and that CRS was associated with asthma, specifically late-onset asthma [7].

In the United States, population-based household surveys carried out by the National Center for Health found a prevalence of self-reported rhinosinusitis of 13% in 2009. The prevalence of CRS was defined as an affirmative answer to the question “In the last 12 months, has the patient had sinusitis diagnosed by a health professional? [16]). A recent study in Sao Paulo, a municipality that has an urban population of 11 million, using a complex two-stage cluster sampling plan, and personal interviews and defining CRS based on the EPOS criteria found a prevalence of 5.51% [15]. The prevalence of doctor-diagnosed using ICD-9 codes as an identifier of CRS in Olmsted, Minnesota reports a prevalence of 2% of the population [17]. In Canada, the prevalence of CRS, defined as an affirmative answer to the question ‘Has the patient had sinusitis diagnosed by a health professional lasting for more than 6 months?’ ranged from 3.4% in male to 5.7% in female subjects, with a response rate of 82% [18]. In a nationwide survey in Korea, the overall prevalence of CRS, defined as the presence of nasal obstruction and nasal discharge lasting more than 3 months together with the endoscopic objective findings such as discolored nasal drainage in the nasal passage or nasal polyps, was 6.95% [8].

## Pharmacoeconomics of CRS

The overall expenditure for CRS has been recognized as a socioeconomic burden, and it includes not only the cost for the medical management of the disease (medication, doctor’s visits, surgery), but also includes costs to the society (medical care systems) and economy (absenteeism and presenteeism, resulting in decreased work productivity). The number of US office-based doctor visits resulting from a diagnosis of chronic rhinosinusitis in 2000 was approximately 11.6 million [19]. Mean medical resource costs were US$921 per patient-year. CRS caused an average of 4.8 days of missed work per 12-month period. The overall yearly economic cost of CRS was US $1539 per patient [20].

In the United States, the national health care costs of CRS in 2007 were estimated as US $8.6 billion per year due to increases in office-based and prescription expenditures (this figure does not include over-the-counter medication or other expenditure by the patient [21]). It has been estimated that antibiotics to treat CRS only may cost more than US $150 million per year [20] and more than 257.000 patients undergo ambulatory sinus surgery per year [22].

## Genetics and epigenetics

Chronic rhinosinusitis (CRS) is a complex inflammatory disease, in which both genetic and environmental factors contribute to its pathogenesis [23]. Based on family studies, it has been estimated that CRS exhibits a heritability of 13% to 53% [24–26], with highest heritability seen in the asthma-nasal polyp-aspirin intolerance triad [25]. Asthma, a disease with an even higher heritability, has been reported to occur in 20% to 31.9% of CRS subjects [27, 28], further indicating the potential genetic component in the etiology of CRS. In addition, the consistent features between nasal polyps/CRSwNP and cystic fibrosis (CF), a common genetic disorder caused by mutations in the CFTR gene, provides extra evidence that genetically determined alterations of sinus mucosal immunology contribute to the development of CRS [29–32]. However, data obtained from studies of CRS in twins are inconsistent [33–35] and suggest that a combination of genetic and environmental factors is likely to play a role in the development of CRS.

Two techniques; namely the candidate gene approach and the genome-wide association study (GWAS) have commonly been employed to investigate population-based associations between specific alleles and certain common diseases. Candidate gene or pathway association studies, which focus on single nucleotide polymorphisms (SNPs) among unrelated individuals characterized by the presence or absence of a phenotypic trait of interest, have mostly been performed in the identification of genes implicated in CRS [36]. These studies mainly exerted great attention on the genes related to innate immunity, relevant regulatory factors, or downstream products involved in the pathogenesis of CRS. An earlier study by Molony et al [37] investigated the association between several human leukocyte antigen (HLA) alleles and CRS and demonstrated that the incidence of HLA-A1B8 was significantly increased in CRSwNP patients with concomitant asthma and aspirin intolerance [37]. Similarly, Luxenberger et al [38] performed a case-control study and reported that HLA-A74 was associated with susceptibility to the development of CRSwNP. Likewise, HLA-DQB1*03 was found to be a risk factor for allergic fungal rhinosinusitis and hypertrophic sinus disease [39]. Although several other HLA alleles, SNPs or haplotypes have also been shown to be associated with CRS in different ethnicity groups [40–45], these studies have been limited by the complicated nature of the genotyping procedures and underpowered statistics.

Although the development of CRS has been reported to be associated with a variety of cytokines (including IL1α [46–48], TNFA [47–49], IL1B [47, 50], IL10 [50, 51], IL33 [52], TGFB1 [53], TNFA1P3 [54], IL4 [55], TSLP [56]), cytokine receptors (IL1RN [57], IL22RA1 [58], IL1RL1 [59], MET [60, 61]), immunity pathways (SERPINA1 [62, 63] TLR2 [64]. IRAK4 [65], NOS1 [66], NOS1AP [66], ALOX5AP [67], ALOX5 [67], CYSLTR1 [67], TP73 [68], COX2 [61]) and airway remodelling related molecules (CACNG6 [69], MMP9 [70, 71], EMID2 [72]), up to now only two polymorphisms in the IL1A (rs17561) and TNFA (rs1800629) genes have been replicated. Karjalainen et al. [46] first reported rs17561 located in exon 5 of the IL1A gene genotyped differently between asthmatics with and without NP in the Finnish population [46]. Similarly, another study demonstrated that IL1A (rs17561), IL1B (rs16944) and TNFA (rs361525 and rs1800629) were associated with susceptibility to CRSwNP in a Turkish cohort [47]. Furthermore, an association between IL1A_rs17561 and the development of severe CRS was shown to be present in a replication study involving 206 Caucasian patients and 106 postal code-matched controls [48]. The SNP rs1800629 in the TNFA gene associated to CRSwNP has also been replicated in two studies [48].

Compared with candidate gene association studies, GWAS do not test any specific hypotheses, but examine the entire human genome to discover any associations between particular genes and disease. However, GWAS of CRS are still lacking due to the high costs involved in genotyping and the large patient cohorts required to ensure sufficient statistical power. One DNA pool-based GWAS has nevertheless determined allele frequencies in separate pools of high density SNP microarray-hybridized DNA from Caucasian CRS patients and control subjects [73]. In this study, the authors identified 600 SNPs from 445 genes with a *P* value < 0.05 among 210 CRS patients and 189 controls; with the top 10 associated genes suggesting a potential role for interactions at the level of the basement membrane and extracellular matrix (LAMA2 and LAMB1), mitochondrial function (PARS2) and lipopolysaccharide degradation (AOAH). More recently, Zhang and colleagues [74] replicated 17 of these CRS susceptibility genes in a Han Chinese population, and demonstrated that at the same SNP locus (rs4504543) an AOAH gene was significantly associated with CRS; thus indicating the potential common genetic basis in the development of CRS in Chinese and Caucasian populations.

Epigenetics is defined as the study of heritable changes in gene expression or cellular phenotype caused by mechanisms other than changes in the DNA sequence. Epigenetic modifications/marks determine how the information in genes is expressed and used by cells, and generally comprise DNA methylation, modification of histone tails and noncoding RNA. Epigenetic mechanisms regulate the expression of key molecules or pathways in immunity and relate those to the pathogenesis of immunologic and inflammatory disorders. It has been suggested that hypermethylation of CpG islands in genes promotes gene silencing. One study investigating the epigenetics of CRS has indicated that activated eosinophils, which are found predominantly in CRSwNP patients, may lead to DNA modifications and gene silencing via 5BrC and aberrant methylation patterns [75]. A recent study examining genome-wide DNA methylation levels in CRSwNP tissues and peripheral blood cells collected from aspirin-intolerant asthma (AIA) and aspirin-tolerant asthma (ATA) patients [76] showed that CRSwNP patients with AIA demonstrated characteristic methylation patterns affecting 337 genes. MicroRNAs are a class of small noncoding RNAs that regulate the target gene expression through effects on mRNA stability and translation. A significant change in gene expression in sino-nasal mucosa from patients with CRS [77] has suggested that CRS may be particularly sensitive to microRNA (miR) regulation. A recent study by Zhang et al [78] reported that there was overexpression of miR-125b in eosinophilic CRSwNP individuals and that this miR played an important role as a regulator of innate immunity via the miR-125b-EIF4E-binding protein 1 in the IFN pathway for mucosal eosinophilia in such patients. Similarly, another study demonstrated that PACT, a protein activator of the interferon-induced protein kinase, associated with the microRNA machinery may be involved in plasma cell function and eosinophilic inflammation in CRSwNP [79, 80].

Although genetic and epigenetic mechanisms contribute to our understanding of the cause and pathogenesis of CRS, and additionally provide an insight into potential future targets and corresponding interventions, CRS remains a complex multifactorial disease, which requires further differentiation. The challenge will be to understand how genetic variation, epigenetic marks and environmental factors interact to lead to the development of specific CRS endotypes.

## Remodeling in CRS

Remodeling is a critical aspect of normal physiology and wound repair in all organs, being defined as ‘modeling again’ or ‘modeling differently’. It is a dynamic process resulting in both extracellular matrix (ECM) production and degradation. This may lead to a normal reconstruction processes with restoration of normal tissue, or may result in pathological reconstruction with formation of pathological tissue [81].

Remodeling in lower airway disease has been extensively studied and reviewed. It includes changes in airway epithelium, lamina propria and submucosa, resulting in airway wall thickening. The main histological features of remodeling are macrophage and lymphocyte infiltration, fibroblast proliferation, angiogenesis, increased connective tissue formation (fibrosis) and tissue destruction. For the upper airway, there is clear evidence that remodeling is also present in chronic rhinosinusitis as well as allergic rhinitis [81–83]. Based on the differential expression of inflammatory cytokines and remodeling patterns, chronic rhinosinusitis with polyp formation (CRSwNP) can be distinguished from chronic rhinosinusitis without polyp formation (CRSsNP).

There are striking differences in the histological appearance of CRSwNP, characterized by albumin accumulation and edema formation, whereas CRSsNP is marked by fibrosis [84]. CRSsNP is typically showing more neutrophilic inflammation than CRSwNP, and also fibrosis formation of the extracellular matrix consisting of excessive collagen deposition and thickening of collagen fibers in the absence of pseudocysts. In contrast, the histology of CRSwNP is typically characterized by the presence of pseudocyst formations consisting of albumin accumulation and edema formation, a lack of collagen within the extracellular matrix and an excessive infiltration of inflammatory cells mainly consisting of eosinophils in about 80% of the Caucasian polyps.

Although Caucasian CRSwNP is characterized by a predominant Th2-type eosinophilic inflammation with high levels of IL-5, ECP and local IgE, and Asian CRSwNP preferentially has a Th1/Th17 polarization signature, typical remodeling features in nasal polyps from both ethnic groups are albumin accumulation and edema (pseudocyst) formation within the extracellular matrix. This questions the link between inflammation and remodeling, as do findings of remodeling in early CRSsNP disease without signs of inflammation [85].

One striking feature is the relative lack of the transforming growth factor beta (TGF- f1) signaling in CRSwNP and consecutively a lack of collagen production [86]. In contrast, CRSsNP is characterized by a mainly Th1-driven inflammation with active TGF-β1 signaling and subsequent excessive collagen deposition and fibrosis formation. TGF-β plays a crucial role in remodeling processes in the airway by the attraction and induction of proliferation of fibroblasts, and the up-regulation of the ECM synthesis [87]. Increases in TGF-β1 protein and mRNA have been measured repeatedly in CRSsNP, in contrast to decreases or lack of increases in CRSwNP [83, 84].

Remodeling is a dynamic process in both health and disease, balancing ECM production and degradation. ECM breakdown is regulated mainly by a family of matrix metalloproteinases (MMPs) and their inhibitors, tissue inhibitors of metalloproteinases (TIMPs). In sinus disease, differing results and a multitude of MMP subtypes make it difficult to interpret data. In CRSsNP, levels of MMP-9 (gelatinase B) and its inhibitor, TIMP-1, are increased, and might counteract each other. Findings of increased concentrations of MMP-9, but not of TIMP-1, in nasal polyps suggest that the MMP-9/TIMP-1 imbalance is associated with ECM degradation in CRSwNP [87]. In addition, MMP-9 was found to be involved in wound healing, and high levels predicts poor healing after sinus surgery [88].

Recent studies also found that the regulation of tissue-plasminogen activators (u-PA and t-PA) might contribute to tissue remodeling and pathogenesis of CRSwNP [89, 90]. However, findings are partially contradicting, which might be due to a lack of correct sub-classification of the disease.

Anti-inflammatory medications (topical steroids being the gold standard) have the potential to suppress inflammation and edema formation. However, studies suggest that anti-inflammatory approaches alone are not successful in reversing changes such as collagen deposition, indicating that early treatment might be crucial for preventing disease progression. Antibiotics such as doxycycline also possess an anti-MMP effect, and have been shown to modify polyp size and healing after surgery [91, 92]. Future studies are needed to optimize the usage of remodeling interventions in disease and after surgery.

The conservative philosophy of endoscopic sinus surgery and minimally invasive sinus technique to relieve ostial obstruction is very likely insufficient in handling severe disease states with high inflammatory loads and/or a dysfunctional mucosa. These patients derive more benefit from maximal surgical options directed toward eliminating the inflammatory load and improving access for topical medication to retard or reverse the mucosal damage. Additionally, removal of irreversibly diseased mucosa allows healthy mucosa to regenerate in its place. Due to the complexity of disease in recalcitrant sinusitis, it is likely that multimodality treatment will serve these patients best [93].

## Epithelial barrier and innate immunity

The sinonasal mucosa serves as the site of interface with inhaled irritants, aero-allergens, commensal organisms and pathogens. Mucociliary clearance and apical junctional complexes (AJCs) between epithelial cells comprise a mechanical barrier between host and environment. Respiratory mucus, which is produced by goblet cells and submucosal glands, traps foreign material and moves it out of the sinuses and nasal cavity towards the nasopharynx. Genetic defects in mucociliary flow are associated with a high incidence of CRS [94, 95]; acquired mucociliary defects and increased mucus viscosity have also been suggested to underlie idiopathic CRS [96, 97]. Sinonasal epithelial cells (ECs), residing beneath the mucus layer, are linked by tight and adherence junctions (AJCs), creating a relatively impermeable barrier. Proteins comprising the AJC are subject to degradation by proteases, such as those found in allergens, bacteria and fungi. In CRSwNP, significantly altered levels of adhesion complex proteins have been identified [98–101] as well as lower levels of intrinsic protective anti-protease activity [102, 103]. Functional studies have also recently suggested that the epithelial barrier is more permeable in nasal polyps [100, 101]. Taken together, these studies suggest that mucociliary dysfunction may play a role in the pathogenesis of CRS broadly, while a porous barrier has been more closely linked to CRSwNP.

Environmental stimuli that breech the mechanical barrier may trigger an innate immune response. Sinonasal epithelial cells (ECs) and other cell types present express pattern recognition receptors (PRRs) that recognize pathogen associated molecular patterns (PAMPs) present on microbes [104, 105]. Cellular damage is detected through damage-associated molecular patterns (DAMPs) [106, 107] and the combined signal of foreign material plus cellular damage governs the release of host defense molecules, cytokines and chemokines. Toll-like receptors (TLRs) are the best-studied PRR, with potential derangements contributing to the development of CRS, but data are thus far inconclusive [105, 108–111]. More recent evidence has indicated that classical taste receptors are also present on ECs functioning as PRRs by detecting microbial products and triggering enhanced mucociliary clearance and release of host defense molecules [112]. Genetic variation in these taste receptors may play a role in CRS disease susceptibility [113].

Nasal ECs secrete a vast arsenal of host defense molecules into the nasal mucus at baseline, with levels augmented upon PRR stimulation [114–116]. These innate responses in ECs are modulated in part by IL-22 and its receptor IL-22R [117, 118] which act in part through the transcription factor STAT 3, broadly mediating mucosal host defense and epithelial repair [119–122]. Decreased expression of some host defense molecules has been associated with CRS [123–127]. While the mechanism for this weakened host defense is unclear, diminished expression of *IL-22R*[128] and blunting of the STAT 3 pathway [129] have been reported in CRS. Regardless, the presence of diminished host defense molecules in CRS suggests the hypothesis that a *primary* sinonasal innate immune defect may contribute to local microbial proliferation fostering the development of CRS in a subset of patients [130].

Beyond host defense molecules, ECs also secrete cytokines and chemokines in response to PRR which foster an inflammatory response and attract and activate innate effector cells [114, 131–136]. In addition, cytokine crosstalk between ECs, innate lymphoid cells (ILCs) and dendritic cells matches the appropriate innate and adaptive response to foreign stimuli. In health, this maintains mucosal homeostasis with (a) tolerance of allergens and commensals and (b) defense against pathogens without the development of chronic inflammation. CRSwNP is characterized by chronic and excessive Th2 inflammation and the specific EC cytokines IL-25, IL-33 and TSLP have been implicated in disease pathogenesis via effects on dendritic cells and type 2 ILCs [127–139]. In support of this hypothesis, large numbers of Type 2 ILCs are present in nasal polyps [140] and high levels of TSLP have been identified suggesting a key role for this cytokine in polyp pathogenesis [141–145]. Levels of other epithelial cytokines with Th2 properties, such as IL-33, have been reported as higher in recalcitrant CRSwNP [146] and genetic studies also suggest that variation near the IL-33 gene is associated with CRSwNP [52]. In regard to IL-25, there is no current evidence for elevated expression or activity of this cytokine in CRS. EC chemokines play a major role in the attraction and activation of innate effector cells including eosinophils, mast cells, neutrophils and macrophages. The tissue changes associated with CRS are presumably secondary to toxic effects of excessive or persistent degranulation of these cell types [6]. In regard to CRS pathogenesis, most interest has centered on eosinophils [147] and mast cells [148] but elevated levels of neutrophils and macrophages are present and phagocytic activity may be impaired in CRS [149].

ECs express enzymes involved in the generation of reactive oxygen species (ROS) and reactive nitrogen species (RNS) that are important in multiple epithelial processes including mucin production, epithelial repair, innate immunity and response to environmental toxins [114, 150]. Variations in activity of these enzyme systems have been proposed to impair barrier function and innate immunity in CRS [151–153] but the clinical significance remains uncertain [154]. EC enzyme systems also likely contribute to tissue levels of eicosanoids, which have been implicated in subtypes of CRSwNP [155–157].

Broadly speaking, CRS has been proposed as a disease characterized by a dysfunctional host-environment interaction at the sinonasal mucosa [6]. While the association of asthma and CRS is well established, the prevalence of other chronic inflammatory disorders in the CRS population was not found to be significantly above background [157]. These observations suggest that host defects in CRS will be centered in the airway mucosa giving rise to the ‘*immune barrier hypothesis*’ which proposes that defects in the coordinated mechanical barrier and/or the innate immune response of the sinonasal epithelium manifests as CRS [158]. Diminished innate host defense coupled with a porous barrier should theoretically lead to increased microbial colonization, accentuated barrier damage and a compensatory adaptive immune response [130]. The ‘immune barrier hypothesis’ does not specifically address the Th subset skewing observed in many CRS subtypes, including the Th2 pattern and B cell infiltrate observed in Western CRSwNP patients. This implies additional, as yet undetermined mechanisms, perhaps centered on EC and ILC signaling, that foster an inappropriate local, adaptive response in the sinonasal mucosa. An excessive and/or inappropriate Th2 adaptive response in this setting may further compromise barrier function and diminish innate immunity, thereby creating a self-perpetuating cycle of disease.

## Pathophysiology: acquired immunity, T cell signatures

T cell patterns, Tregs, follicular structures, immunoglobulins.

The acquired immune system consists of T cell and B cell subsets, armed with different abilities to fight pathogens and orchestrate inflammation. CRSsNP and CRSwNP are characterized by specific compositions of lymphocytes; and specifically T helper cell signatures do have an important impact on the type of mucosal inflammation with respect to neutrophilic vs. eosinophilic predominance. Today, we recognize several Th cell signatures in CRS, with Th1, Th2 and Th17 cells being most prominent [159, 160]. Pilot studies in upper airway mucosal tissue pointed to the fact that interferon-gamma producing Th1 cells were characteristic for CRSsNP, whereas interleukin-5 producing Th2 cells were typically found in CRSwNP [159]. Studies together with Chinese colleagues revealed later that the picture is much more complex, with Th1, Th2 and Th17 cells coexisting in the airway mucosa, and that the relative distribution of those cells would differ greatly between continents [160]. In Caucasians, more than 80% of polyps express a Th2 profile, whereas in China, polyps express a predominant Th17 cell profile. However, Caucasian nasal polyps in cystic fibrosis patients do also mostly show a characteristic Th17 signature [161]. Recent studies showed that the signature of Th cells may be different from one to the next individual, demanding a much more specific investigation into clusters of T cells orchestrating the inflammation in subgroups of patients, which may be associated with very different co-morbidity profiles [162] (see also chapter on cluster analysis). Furthermore, Asian more than Caucasian polyps may not show a dominant T cell at all, underlining the dissociation between remodeling and inflammatory patterns in nasal polyp disease [163].

Apart from T helper cells, the presence and functionality of T regulatory cells differentiates CRSsNP and CRSwNP in that a deficit of Tregs has been described in nasal polyps, but not in CRSsNP [164, 165]. An impaired migration of Treg cells and a deficit in TGF-beta formation has been claimed to possibly cause this observation [164, 166]. It is reasonable to appreciate that a deficit in Foxp3 expressing Tregs could account for the persistence of inflammation observed specifically in CRSwNP, although this needs to be tested. Recent evidence has pointed to suppressor of cytokine signaling3 (SOCS3) protein, overexpressed in CRSwNP by dendritic cells and Tregs, as a candidate for therapy, as SOCS3 suppresses the expression of Foxp3 [167].

Recently, also IL-21 and IL-21-producing T follicular helper (Tfh)-like cells were identified as an increased population in nasal polyposis, with the staphylococcal superantigen SEB being one of the triggers for IL-21 expression [168]. The authors speculate that T-follicular helper cells and their product, IL-21, are important in the pathophysiology of nasal polyposis by stimulating local immunoglobulin production and germinal center formation.

Apart from an increase in T cells, also the numbers of B and plasma cells are significantly up-regulated in CRSwNP [159], suggesting a highly activated local immunoglobulin production. The overproduction of B cell-activating factor of the TNF family BAFF and other plasma cell differentiation molecules may partially account for this up-regulation [134, 169]. There is clear evidence for local receptor revision and class switching in CRSwNP, but not CRSsNP, and the expression of inducible lymphoid tissue, follicle-like structures, has been demonstrated in the airway mucosa of polyps [169], as well as the expression of all necessary regulators of class switch recombination including AID, RAGs and others [169, 170]. Specifically nasal polyps thus should be understood as an immunoglobulin generating disease, the role of which is not completely understood so far.

## Immunoglobulin synthesis

Although the etiology of the inflammation associated with CRS is not completely understood, the presence of bacteria within the nose and paranasal sinuses is well documented [171, 172]. Yet there is much diversity on the type of pathogens identified primarily due to the point in time, manner and mode of sample collection, treatment methods used and techniques of bacterial culture. In CRS without underlying infection, bacterial colonization is considered to exacerbate a noninfectious inflammatory response via bacterial allergic mechanisms. Bacteria- specific IgE has been reported in 57% of patients with CRS as compared to only 10% in subjects with allergic rhinitis [173] and bacteria like Staphylococcus aureus possess the ability to elicit exotoxins, and superantigens can activate subpopulations of the T-lymphocytes (5-30%) [174]. Bacterial superantigens in the pathogenesis of CRSwNP, superantigen production, and host T-lymphocyte response are crucial components of common chronic eosinophilic-lymphocytic respiratory mucosal disorders [175] and staphylococcal superantigen-specific IgE antibodies to the superantigens SEA and SEB have been detected in nasal polyp tissue [176].

In CRSwNP, there is an increase in the Th2 cytokines like IL-4, IL-5 and IL-13 [177, 178] and the intensity of eosinophils in the tissues of these patients is markedly increased in the presence of co-existing asthma or positive allergy skin tests. The increased presence of IL-4 and IL-13 can play a role in upregulating VCAM-1 and thus facilitates the further infiltration by eosinophils. IL-4, IL-13 and TNF-alpha from mast cells and T cells can upregulate eotaxin production in epithelial cells [178]. Immunoglobulins like IgA, IgE, IgG and IgM are also increased in polyp fluid and tissue [177] and the concentrations of total IgE, IL-5, eotaxin, ECP, LTC_4_/D_4_/E_4_, and sCD23 were significantly higher in nasal polyp tissue as compared with non-polyp tissue [180]. Total IgE correlates significantly with IL-5, ECP, LTC_4_/D_4_/E_4_, and sCD23 and with the number of eosinophils in nasal polyps [176, 180]. In fact, IgE and IgA producing plasma cells are particularly prominent within NP and the locally produced IgE and IgA are potentially involved in activation of mast cells and eosinophils, which in turn contribute to inflammation in these tissues [181]. Total IgE levels in NPs are often highly increased, independent of atopy. Specific IgE to SEs usually can be found locally within the mucosa but not necessarily in the serum [180–182]. Follicle-like structures can be identified frequently in NP tissues, which highly express IgE antibodies binding to SEs [184]. In addition to IgE, NPs have increased IgA levels [182]. The observation of increased local immunoglobulin production is supported by the expression of the immunoglobulin diversification enzyme activation-induced deaminase, indicating local immunoglobulin class-switching to IgE and IgA [170]. Local IgE antibodies, although polyclonal and directed against a range of inhalant and SE-related allergens, are functional and capable of degranulating mast cells [184] and activated mast cells release histamine and tryptase which upregulate the production of RANTES and GM-CSF from epithelial cells thus facilitating eosinophil infiltration and survival [177, 184–189]. In fact, increased levels of tryptase and histamine (exceeding levels of 4000 ng/ml) have been found in nasal polyps and a good correlation between the levels of ECP and histamine and tryptase is also documented [178–190]. Studies have shown local production of IgE in the allergic nasal mucosa, and that mast cells can drive this IgE production in B cells further enhancing the inflammation [191]. As mast cells can be activated by polyclonal IgE; such a scenario can further enhance the local inflammation. Furthermore, a deficiency of Treg cells and increase in TSLP in nasal polyps (144) may play an important role in the enhancement of the severity of Th2 inflammation in nasal polyps and the persistence and growth of NP [164, 165]. More recently, Gevaert et al demonstrated RAG1 and RAG2 mRNA concentrations are increased in CRSwNP and correlated with the magnitude of inflammation and the presence of *S. aureus* enterotoxin (superantigen)-specific IgE in the nasal polyp mucosa confirming local receptor revision and class switching to IgE, and B-cell differentiation into IgE-secreting plasma cells in CRSwNP [169].

The presence of SE-IgE antibodies and the increase in local IgE suggests an association with the comorbidity of asthma [162, 192]. More recently, we (Kimura et al) have demonstrated increased expression of Thymic Stromal Lymphopoietin (TSLP) in nasal polyps irrespective of the atopic status, as compared to the allergic nasal mucosa and the TSLP expression was in good correlation with eosinophils and IgE in the nasal polyp [144]. The role of IgA in the pathology of CRS is unknown, but the presence of IgA in patients with most types of chronic mucosal inflammation, such as periodontitis, suggests that IgA is important and might identify a unique endotype of CRS [193]

**Figure 2:**
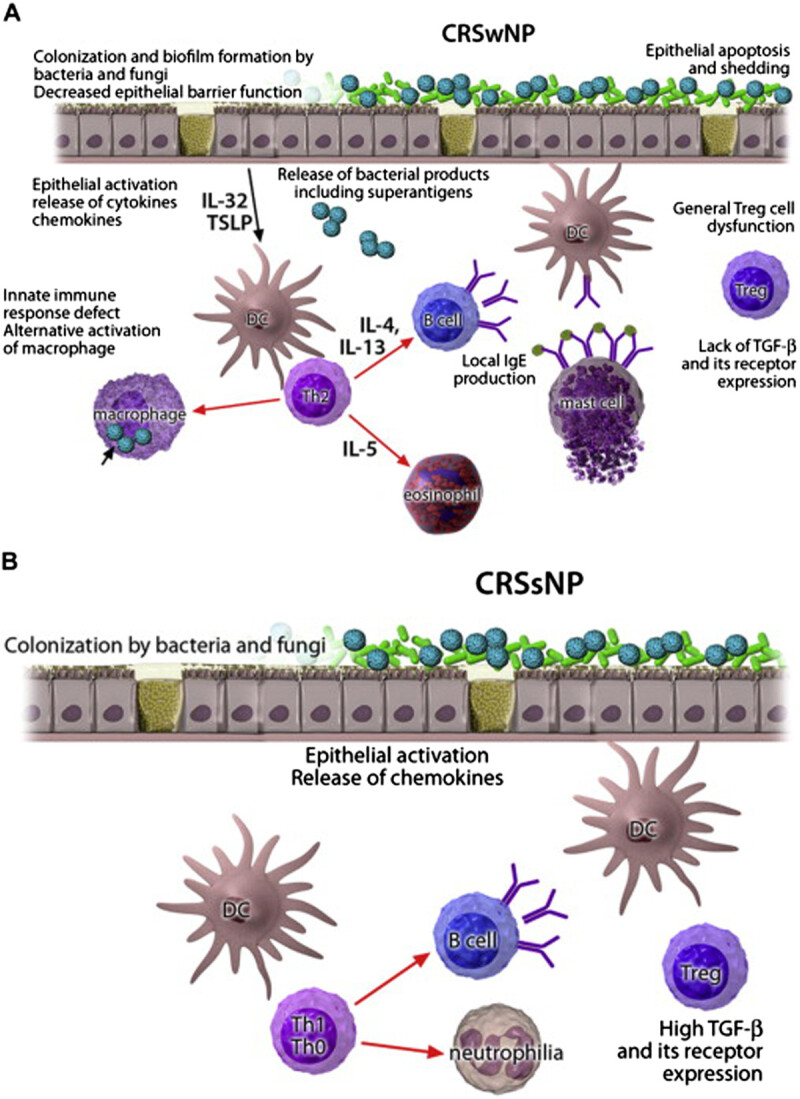
**Pathomechanisms of CRS. A**. CRSwNP. In a TH2-type microenvironment with general lack of regulatory T (Treg) cell function, IL-5 induces eosinophilia, and IL-4 and IL-13 induce local IgE production. An alternatively activated macrophage subset contributes to the inflammation. The activation of epithelium colonized by bacteria and fungi leads to release of proinflammatory chemokines and cytokines with increased thymic stromal lymphopoietin (TSLP) and IL-32 levels. Activated epithelial cells die, with apoptosis resulting in a compromised epithelial barrier. **B**. CRSsNP. Instead of a TH2-skewed T-cell response, a TH1 or a mixed TH0 response predominates, neutrophilia is often associated, and expression of TGF-β and its receptors is increased. DC, Dendritic cell. This figure is reused with permission from the Journal of Allergy and Clinical Immunology [198].

Recent findings point to superantigens as possible causal agents in the intrinsic form of severe asthma, and an anti-IgE strategy has shown promising therapeutic potential in nonatopic patients with nasal polyps and asthma [194]. These findings should lead to a clinically relevant endotyping of patients with upper and lower airway disease and to a new understanding of the role of IgE 'above atopy'. A proof-of-concept study demonstrated the functionality of local polyclonal IgE in the airways by analyzing the effect of anti-IgE therapy in inducing a substantial decrease in total polyp scores after 16 weeks in the omalizumab group as compared with baseline values by computed tomographic (CT) scanning. In addition, Omalizumab significantly improved upper and lower airway symptoms (nasal congestion, anterior rhinorrhea, loss of sense of smell, wheezing, and dyspnea) and asthma-related quality-of-life scores [195]. The demonstration of direct IgE switching and the existence of cellular IgE memory suggest the possibility of targeting these mechanisms for the treatment of IgE-mediated diseases [196, 197]. A recent A PRACTALL document of the European Academy of Allergy and Clinical Immunology and the American Academy of Allergy, Asthma & Immunology has summarized the endotypes and phenotypes of CRSwNP and CRSsNP [198] (Figure 2). More research is required to clarify if CRS characterized by local IgE production can be considered a CRS endotype.

## Pathophysiology - infections, microbiome

Universal to the pathophysiology of chronic rhinosinusitis (CRS) is the persistence of inflammation. The cause of this inflammation in an individual patient, however, is most often indiscernible [199]. An infectious etiology for CRS has long been postulated [200]; although there is significant scientific evidence that acute rhinosinusitis is primarily an episodic viral infection of the paranasal sinuses, the scientific evidence for infection as a significant component in the pathogenesis of CRS is less clear. Acute exacerbations of CRS are likely due to infectious exacerbations secondary to viral infections. Although a causal relationship has yet to be established between bacterial infection and CRS, some studies [201, 202] strongly implicate bacterial infection. An interesting study examined the microbiology of sinus aspirates during the transition from acute rhinosinusitis to CRS [201]. Patients in the study had failed to respond to antibiotic treatment and had sequential cultures performed over a 5- to 7-week period after the initial acute infection. Initially, typical bacteria for acute rhinosinusitis were recovered, including *Streptococcus pneumoniae, Haemophilus influenzae*, and *Moraxella catarrhalis*. As patients transitioned to the chronic phase, a mixed bacterial infection prevailed involving anaerobic agents such as *Prevotella, Fusobacterium,* and *Peptostreptococcus* species. This data provides evidence that bacteriology in CRS is different from that of control patients before acquisition of CRS. It is fair to say that bacteria are encountered frequently in the setting of CRS; the flora of the paranasal sinuses seems to be altered, with a higher prevalence of *Staphylococcus* species and anaerobes. This flora is often polymicrobial and may exhibit significant antibiotic resistance. Also, a study which examined the microbiology of recurrent infectious rhinosinusitis after endoscopic sinus surgery has been reported [202]. The data support bacterial infection as causing symptoms even after endoscopic sinus surgery, further implying that bacteria even in patent sinuses may cause symptoms. Therefore, it is highly likely that bacteria are involved at least to some degree in the pathogenesis of CRS. The magnitude of that involvement has yet to be determined [203].

Varying levels of involvement have been proposed, including direct bacterial infection causing inflammation and symptoms of CRS, bacterial by-products leading to mucosal injury, and hypersensitivity to bacterial antigens [203]. CRS can occur with or without nasal polyps, and accumulating evidence now identifies *Staphylococcus aureus* superantigens contributing to CRS with nasal polyposis [181]. Superantigens are thought to affect multiple cell types and skew the cytokine response towards a Th2 phenotype including both eosinophilia and the production of polyclonal IgE, which in turn could be further linked to asthma [162, 204]. It remains unclear why superantigen effects can be demonstrated in only approximately half of the cases of CRS with nasal polyposis; hence, *Staphylococcus aureus* superantigens are generally seen by many as disease modifiers rather than discrete aetiologic agents [156].

Bacterial biofilms have also been implicated in CRS aetiology and pathogenesis. Biofilms are highly organized structures composed of communities of bacteria encased within a protective extracellular matrix. The formation of bacterial biofilms on surfaces such as the sinonasal mucosa reflects a universal strategy for survival in conditions less than optimal for growth [205, 206]. Biofilms serve to protect bacteria from both host defense and antibiotics [207] and are believed to be a source of recurrent exacerbations in CRS through the periodic release of free-floating planktonic bacteria [208]. Multiple studies have confirmed the presence of bacterial biofilms in the sinonasal cavity of patients with CRS using operative samples. Bacterial biofilms have been detected by various methods in 30-100% of these samples [209]. Multiple bacterial species have been associated with CRS biofilms including *Haemophilus influenzae*, *Staphylococcus aureus*, *Streptococcus pneumoniae*, *Moraxella catarrhalis*[205, 206, 210–212]. In particular, *Staphylococcus aureus* has been associated with a particularly poor prognosis [213]. It has been suggested that *Staphylococcus aureus* biofilm fosters a Th2 adaptive immune response independent of any *Staphylococcus aureus* superantigen effect [214]. Recent studies suggest that disruption of the host epithelium may permit biofilm mediated inflammatory effects on the sinonasal tissues [215]. It is widely accepted that biofilms are a bacterial adaptation facilitating resistance to host defenses and antibiotics, helping to foster recalcitrant diseases. Moreover, it is also possible that biofilm directed therapies will prove useful in the management of CRS. However, it remains much less clear whether biofilms have any role in the initial establishment of CRS [216].

Several recent studies have implicated fungi in the pathogenesis of CRS. However, the role of fungi in CRS has generated much controversy in the last decade [217, 218]. Sensitive detection techniques have shown the presence of fungi in close to 100% of both CRS patients and controls [219, 220]. However, as opposed to controls, eosinophils were exhibited in the nasal tissues and lumen of CRS patients, with no evidence for IgE mediated mould allergy according to serum data [219]. These observations formed the basis of the “Fungal Hypothesis of CRS”, which proposed that an excessive, non-IgE mediated host response to common airborne fungi is the primary pathogenic trigger in most form of CRS, both polypoid and non-polypoid, varying only in intensity [221–223]. The primary evidence cited to support this theory was the relative hyper reactivity of peripheral blood mononuclear cells (PBMC) from CRS patients in response to stimulation with supra-physiologic doses of *Alternaria* antigen *in vitro*[224]. Significantly higher levels of Th1 and Th2 were found in the PBMCs of CRS patients after exposure to *Alternaria* extract, leading some to believe that this heightened response reflected an immunologic sensitization of T cells to *Alternaria* and therefore was seen as a possible cause of the CRS inflammatory response. As further evidence, nasal mucus or tissue from CRS patients triggered eosinophil migration [225] and a 60-kDa component of the *Alternaria* fungus was later shown to trigger eosinophil degranulation via PAR receptor activation *in vitro*[226]. The effector role for eosinophils against fungi was proposed even though eosinophils do not normally participate to a significant degree in the host defense response targeting fungal organisms [227]. Further challenges to the “Fungal Hypothesis” included the observation that the majority of patients in these studies [224, 225] had concomitant asthma, and the heightened cytokine responses from PBMCs as well as the eosinophil migration may reflect priming by asthma rather than CRS [217, 218]. Also, other investigators failed to replicate the fungal-induced cytokine responses from PBMCs, proving there was clear universal hyper-responsiveness to fungal antigens in CRS patients [228, 229]. Despite these findings, a series of drug trials using intranasal anti-fungal agents was introduced that initially provided mixed support [230–233]; finally, an extensive, multicenter, blinded, randomized trial using intranasal amphotericin failed to show any evidence of efficacy [234]. More significantly, a follow up study indicated that amphotericin had no significant effect on any pro-inflammatory chemokine, cytokine or growth factors in the CRS lavage samples [235]. Overall, the current literature does not support the routine use of topical anti-fungals for CRS [236] and there is little support for the originally proposed fungal hypothesis.

Microbiome research is one of new perspectives in human health research, including CRS. Studies on the total genomes of bacteria inhabiting the body, termed the microbiome, have recently been performed at National Institutes of Health [237]. This Human Microbiome Project (HMP) demonstrated the presence of more than 10,000 microbial species in the human body, and it was estimated that 81-99% of the total microbial species inhabiting healthy individuals were identified. The association between CRS and bacteria in the nasal cavity and paranasal sinuses has been investigated in many studies, but gene analysis of these bacteria to identify the microbiome has only been performed in a few studies [237–241]. The first reported study was performed in 2003, in which a bacteria-specific gene, 16 s rDNA, was amplified from the mucosa and maxillary sinus lavage of 11 patients with maxillary sinusitis. Bacterial genes were amplified in 4 patients and identified as *Staphylococcus aureus*, gram-positives, -negatives, and anaerobes. However, no fungus was detected. In 2010, bacterial gene analysis was performed using the mucosa. Bacterial genes were amplified in all 18 patients, and *Staphylococcus aureus* and coagulase - negative *Staphylococci* (CNS) was detected in many samples along with anaerobes. The involvement of anaerobes in the development of CRS has been suggested, and this study demonstrated it at the gene level. In another study, maxillary sinus lavage was analyzed, and a total of 142 bacterial genes were amplified including many genes of indigenous bacteria in the oral cavity. In a recently reported study, cotton swabs of 15 CRS patients were analyzed, and more than 50,000 bacterial genes were detected in total. It was pointed out that the incidence of asthma and the dominance of *Staphylococcus aureus* increased as the diversity of bacterial genes in the samples decreased. Questions remain with regard to the relationship of CRS with the microbiome in the nasal cavity and paranasal sinuses, also due to possible contamination and inconsistency of the analytical methods. Since the recent progression in analytical methods has facilitated the investigation of the microbiome, further studies of the relationship between CRS and the microbiome is warranted.

## Clinical manifestation, diagnosis, and differential diagnosis: adults

The diagnosis of chronic rhinosinusitis (CRS) is based on the presence of two sinonasal symptoms in association with clinical or radiological evidence for sinonasal inflammation. Two of the following symptoms should be present: nasal secretions, nasal obstruction, facial pain, or smell dysfunction. CRS has long been considered to represent a heterogeneous collection of diseases.

Nasal polyposis is a subtype of chronic rhinosinusitis; the term “polyp” refers to outgrowths of tissue into the nasal cavity. The most common site of origin of nasal polyps is the anterior ethmoid region. Nasal polyps cause nasal obstruction, hyposmia, recurrent infection, and impaired quality of life. Twenty to 90% of patients with nasal polyps have asthma. The large range reflexes both the severity of the asthma, age of the patient and the presence of aspirin sensitivity. When evaluating someone for nasal polyps one should question about the presence of lung disease.

### Diagnosis

A negative physical examination can eliminate serious diseases that mimic CRS.. Unilateral otitis media with effusion can signal a mass in the nasopharynx. Facial numbness raises concern about a sinus malignancy. Facial swelling or interference with ocular movements portends a complication of rhinosinusitis. Fever or enlarged neck lymph nodes are not associated with routine CRS. Whereas most sinusitis is rhinogenic in nature, dental infections can initiate sinusitis. Purulent-appearing post nasal discharge is a strong sign of a sinus infection.

The most common symptom is nasal obstruction. Hypoxia, hypercapnia, snoring, sleep disorders, and an increased risk of hypertension may develop in patients with nasal polyposis. Polyps may obstruct airflow to the olfactory cleft and lead to loss of the sense of smell [242]. Nasal polyps are uncommon in children, and their presence should prompt evaluation for cystic fibrosis. A unilateral nasal polyp should raise the suspicion of an inverted papilloma or tumor in adults, or of dermoid cysts, encephaloceles, and gliomas in children. Examination of the oral cavity may show polyps behind the free margin of the soft palate in cases of antrochoanal polyps or postnasal drips that are related to coexisting sinusitis.

Nasal endoscopy provides excellent visualization of polyps, especially of small polyps in the middle meatus. It also shows nasal polyps originating from contact areas in middle meatus and nasal anatomic abnormalities. Culture of the discharge and a biopsy can be performed under endoscopic guidance. Cultures from the middle meatus or osteomeatal complex area have been shown to correlate with cultures obtained from within the sinuses.

Computed tomography (CT) is the preferred technical tool for confirming inflammation in the paranasal sinus cavities. It shows the extent of disease. Caution should be taken in the interpretation of the CT scan abnormalities, as asymptomatic individuals may have CT scan abnormalities in the sinonasal cavities and a common cold or exposure to allergen in an individual with allergic rhinitis may induce major inflammatory changes in all sinus cavities. The timing of a CT scan and the correlation between symptoms and CT scan abnormalities are crucial for a correct clinical diagnosis. A CT scan is essential in cases of unilateral disease, suspect sinister pathology, failure of medical treatment, and when complications are suspected. CT is best performed after medical management for delineation of the chronic disease component. CT is superior to magnetic resonance imaging (MRI) in the depiction of bone details. MRI is advisable when skull base erosion is noted adjacent to an area of sinus opacity. It can differentiate between sinus disease eroding the skull base and a meningocele or encephalocele. MRI also helps to differentiate a tumor from secretions retained secondary to obstruction by the tumor.

Other investigations include allergy testing, a pulmonary function test, biopsies, a sweat chloride test, smell test or genetic testing for detection of cystic fibrosis, aspirin intolerance testing, swabs and fungal stains and cultures. Skin testing does not always accurately confirm a role for allergens in the absence of a definitive medical history or positive nasal provocation test.

### Differential diagnosis

The differential diagnosis can be divided into diseases that mimic CRS and into diseases associated with CRS. The differential diagnosis of CRS includes perennial and persistent allergic rhinitis, nonallergic rhinitis, immotile cilia syndrome, immunodeficiency, hormonal rhinitis, drug-induced rhinitis, structural abnormalities, adenoidal hypertrophy, and tumors. Conditions associated with nasal polyps include asthma, aspirin intolerance, allergic fungal rhinosinusitis, Churg-Strauss syndrome, and cystic fibrosis. There is still an ongoing debate to as what is the precise role of microorganisms in chronic rhinosinusitis. Biofilms as well as mucosal immune responses to microbes may be involved in the pathology of CRS.

CRS maybe aggravated by anatomic deformities like nasal septal deviations, septal perforations, and nasal valve dysfunction. A clear nasal discharge may be a manifestation of rhinitis, or of posttraumatic leakage of cerebrospinal fluid, whereas colored secretions are often encountered in the case of infectious exacerbations of rhinosinusitis. Smell dysfunction, ranging from hyposmia to anosmia and parosmia, often represents a key symptom of nasal polyps, but is also found in neurodegenerative diseases or intracranial lesions, or it may be congenital. Facial pain or headache may have an odontogenic, vascular, or neurologic origin.

Besides the differential diagnosis, one should bear in mind that rhinosinusitis represents a complex pathology, with many factors influencing the severity of the disease. Any underlying sensitization to inhalant allergens may give rise to an allergic immune response that aggravates the inflammation in CRS. Environmental factors, including cigarette smoke, as well as occupational factors may contribute to the disease process in CRS. These factors should be taken into account when a patient with CRS is evaluated, as diagnostic neglect of these conditions may lead to suboptimal treatment [3, 6, 243–245].

## Specific aspects in children

Pediatric rhinosinusitis is an important medical problem. It is estimated that 5-10% of upper respiratory tract infections (URI) in children are complicated by acute rhinosinusitis (ARS) and 6-13% of all children develop rhinosinusitis by the age of 3 years [246].

Of interest, the immunopathology of rhinosinusitis in children may be distinct from adults. Chan et al. studied the histopathology of young children (median age 3.0 years) and revealed a predominant lymphocytic infiltration instead of the typical eosinophilic infiltration found in adult rhinosinusitis. They also demonstrated less submucosal glandular hyperplasia and thinner epithelium and basement membrane in pediatric compared to adult rhinosinusitis [247]. These findings were confirmed in a recent study which showed more epithelial shedding, tissue eosinophilia and extensive fibrosis in CRS in adults than in children [248]. It is unclear, however, whether this is just a reflection of age or duration of disease, and disease phenotypes are comparable.

### Predisposing factors leading to rhinosinusitis in children

Adenoidal hypertrophy, URI and allergic rhinitis (AR) are more common in children than in adults. A recent study found that adenoids removed from children with CRS had dense mature biofilms covering 95% of the mucosal surface (versus 1.9% in controls) [249]. Of note, chronic maxillary sinusitis and chronic otitis media with effusion can occur concurrently in children and often share the same organisms (in 69% of culture positive patients)[250]. In recurrent rhinosinusitis, the organisms are likely to be resistant organisms [251]. Rhinosinusitis caused by fungal infection is not common in children. The underlying immunological defect should be searched if such condition occurs [246].

Rhinosinusitis may also be a manifestation of gastroesophageal reflux (GERD). The acid reflux may cause inflammation in the nasopharynx leading to mucosal swelling and sinus ostial obstruction [252]. The treatment of GERD by using histamine-2 receptor antagonist and/or proton pump inhibitor may improve the symptoms of sinusitis in children [2].

Anatomical abnormalities are not common in children [252–254]. Retention of secretion due to mucociliary dysfunction can be found in immotile cilia syndrome (Kartagener syndrome) and cystic fibrosis [252, 253]. The immunodeficiencies most frequently associated with rhinosinusitis are B cell defects such as hypogammaglobulinemia, agammaglobulinemia, IgG subclass deficiency, common variable immunodeficiency and selective IgA deficiency. However, severe T-cell dysfunctions or immunosuppressive agents can lead to recurrent or severe rhinosinusitis. Furthermore, defects of innate immunity such as collectin, antimicrobial peptide or Toll-like receptor defects can facilitate rhinosinusitis [252].

### Clinical manifestation of rhinosinusitis in children

Children with rhinosinusitis can present with similar symptoms as adults, but decreased sense of smell or facial pain are less common while cough is more common [6, 253].

### Investigations

CT scan of the sinuses is recommended in severe disease or complications when surgical treatment might be indicated [2, 13]. Sinus aspiration and culture is recommended in rare cases which do not respond to medical treatment, immunodeficiency, severe disease with complications or nosocomial infection [13, 255].

Other investigations are considered to define predisposing factors leading to CRS [2]. To exclude AR, skin prick test should be done. If immunodeficiency is suspected, measuring of IgG, IgA, IgM and IgG subclass as well as assessing the specific antibody response to pneumococcal vaccines are helpful. In immotile cilia syndrome, the suggestive history includes frequent rhinosinusitis, otitis media or pneumonia. Half of these patients have situs inversus and/or dextrocardia. The saccharin test and biopsy of inferior turbinate to examine the cilia under electron microscope could confirm the diagnosis. In cystic fibrosis, patients may present with a medialization of the lateral nasal wall, nasal polyps, *Pseudomonas aeruginosa or Staphylococcus aureus* infections in early childhood; the sweat chloride test is helpful, and genetic investigations confirm the diagnosis [2].

### Diagnosis

The European Rhinologic Society has developed a European Position Paper on Rhinosinusitis and Nasal Polyps (EPOS) in 2007 and updated in 2012 [6, 13]. They suggested that the criteria for the diagnosis of CRS in children included the following:

1. Symptoms persist for ≥ 12 wk with ≥ 2 symptoms, one of which should be either nasal blockage/obstruction/congestion or nasal discharge (anterior/post nasal drip);
2. ± facial pain/pressure,
3. ± cough;

In addition, allergy evaluation should be performed in cases with suggestive history. CT scan is not recommended unless surgery is considered.

### Differential diagnosis

A foreign body in the nose should be suspected when small children present with unilateral, foul-smell nasal discharge. Adenoidal hypertrophy causes symptoms close to CRS [2]. However, nasal congestion, snoring and hyponasal voice are more common in adenoidal hypertrophy than in CRS [13].

### Complication

In young children, orbital complications in acute ethmoiditis or maxillary sinusitis are more common than in adults because of the thin bony walls between sinuses and the orbit [13, 246].

### Management

The management scheme for CRS in children according to our practice and to the EP3OS guideline is not much different from adults, however adenoidectomy and sinus irrigation should be considered before surgery in case of young children. Ungkanont et al. reported that adenoidectomy reduced rhinosinusitis episodes especially in children with obstructive symptoms of the upper airway [256]. A meta-analysis supported that adenoidectomy decreased symptoms in 70% of CRS children [257].

### Prevention in patients at high risk

1. Appropriate treatment of rhinosinusitis with antibiotics according to the guidelines, if unavoidable [258].
2. Decreased exposure to infection sources such as avoid day care attendance, frequent hand washing and avoid ill-contact [3].
3. Pneumococcal vaccine can decrease the incidence of otitis media and pneumonia in children [259]. However, similar evidence for prevention of rhinosinusitis is lacking.
4. Treatment of the underlying factors, if possible [258].
5. Antibiotic prophylaxis may be considered in CRS. Wallwork et al. provided roxithromycin to adults with CRS compared to placebo for 3 months and found that the treatment group had less symptoms of rhinosinusitis and increased mucociliary function [260]. In children, we performed a retrospective study using azithromycin for the prevention of recurrent sinusitis and found a 50% reduction of sinusitis episodes at 12 months of 70% [261]. A prospective, randomized, double blinded, placebo controlled study is ongoing.

## Natural course of disease

CRS is phenotyped as CRS without (CRSsNP) and with nasal polyps (CRSwNP), based on symptoms (loss of smell is typical for CRSwNP, headache and facial pain are typical for CRSsNP) and nasal endoscopy (bilateral presence of polyps in the nasal cavity) [1]. Knowledge about the natural course of these diseases is currently limited. It is unclear whether periods of acute rhinosinusitis normally precede the onset of chronic sinus disease, or a single episode is sufficient to initiate CRS, and how the onset of disease differs from CRSsNP to CRSwNP. It also is unknown which symptoms are the first to appear, and the exact length of time until the disease is diagnosed and treated. So far, cigarette smoking is the only identified risk factor for CRS [9]. However, there is a lack of understanding of pathomechanisms and predictors for disease recurrence, the impact of co-existing allergic disease, and the development of co-morbid asthma or aspirin sensitivity. Furthermore the precise dynamics of levels of inflammatory mediators in the natural course of disease, in particular during acute exacerbation and exacerbation-free interval as well as within the framework of the different phenotypes and endotypes need to be better understood.

A recent Europe-wide epidemiologic study on the prevalence of CRS demonstrated a clear increased risk to suffer from late-onset asthma for CRS patients [7]. Within CRS, there is evidence that Th2-biased CRSwNP has a clearly increased risk of asthma co-morbidity in Caucasians [162], whereas CRSsNP does not significantly impact the development of asthma, but is associated with other lower airway disease [6]. The prominent predicting factor for asthma co-morbidity in nasal polyps is IgE to staphylococcal superantigens, SEs [162]; as there is recent evidence that IgE to SEs is also associated with asthma Europe-wide [262], and specifically has been linked to severe late-onset asthma [263], it is likely that IgE to SEs may play an important role in the expansion from upper to lower airway disease.

Current initiatives to further differentiate CRS based on endotypes (specific pathophysiological principles such as Th2 driven inflammation) as well as the role of the microbiome [264] will allow for better differentiating the course of disease in the near future. This then will facilitate an estimation of the severity of disease and the socio-economic burden to an individual and society.

## Co-morbidities and contributing factors-asthma and AERD

Asthma commonly co-exists with CRS [5]. Whether chronic sinus disease causes asthma is debatable, it is however clear that there is a strong link between the two conditions. Connecting these conditions may be related to neural pathways, which can trigger the release of inflammatory mediators; though less likely is the role of micro-aspiration from the upper to the lower airways [265]. Another possibility is a systemic immunologic cross talk between the upper and lower airways [266]. There is evidence that markers such as interleukin-5 and SE-IgE within the nasal polyp tissue are associated with co-morbid asthma, supporting the idea of a systemic immunologic cross talk) [162]. Data from a 12 country European study showed a strong association of asthma with CRS (adjusted OR: 3.47; 95% CI: 3.20-3.76) at all ages [7]. This survey also found that CRS in the absence of nasal allergies was positively associated with late-onset asthma.

Studies show a close relationship between the severity of CRS and asthma. An Italian study found the frequency of rhinosinusitis was the same in patients with mild-moderate vs. severe corticosteroid-dependent asthma, but sinus clinical symptomology and severity of sinus disease documented by CT scan was significantly worse in the severe corticosteroid-dependent asthmatics compared the mild-moderate group [267]. A US study also found increasing severity of asthma is associated with advancing radiological severity of CRS [268]. Ten Brinke et al show a direct connection between mucosal thickness in the sinuses and bronchial inflammation in severe asthma, particularly in patients with adult-onset disease [269]. Also histopathological findings of asthma, including epithelial shedding and basement membrane thickening features of airway remodeling, eosinophillic infiltration, T helper cell involvement and interleukin 5 production, as well as IgE formation are present in CRS and asthma [270, 273], suggesting similar pathological processes in CRS and asthma.

Aspirin-exacerbated respiratory disease (AERD) is a clinical syndrome, which results in CRS with nasal polyps and asthma and is exacerbated after ingestion of aspirin (ASA) and non-steroidal anti-inflammatory drugs (NSAID) [271]. This condition used to be called “aspirin triad” but now is labeled AERD as the main underlying problem here is a chronic inflammatory disease and not drug hypersensitivity as it is only occasionally triggered by ASA or NSAID. It tends to start in adulthood between 20 and 40 years of age. These aspirin sensitive asthmatics have a much higher rate of nasal polyps compared to asthma patients that are aspirin tolerant and have a much more severe and protracted course with their CRS [272]. Classically nasosinal symptoms appear prior to asthma followed by the development of nasal polyps. This entity can be confirmed by ASA challenge testing. The pathology appears to be related to an imbalance between the cyclooxygenase (COX) and lipoxygenase pathways leading to overproduction of COX-1, which results in increased production of cysteinyl leukotrienes. Also there is probably a role for cytokine production such as IL-5 and staphylococcal superantigens amplifying the inflammatory course. Treatment is difficult in these patients usually requiring repeated surgeries related to the nasal polyposis and aggressive asthma therapy with inhaled and/or oral corticosteroids and other agents. Aspirin desensitization may be beneficial in some of these patients.

## Co-morbidities and contributing factors – smoking, brochiectasis and COPD

Tobacco smoke exposure is considered an important negative prognostic factor for chronic rhinosinusitis (CRS), and smoking has been demonstrated to increase the risk for CRS [9]. Tobacco smoke is composed of a complex mixture of over 5000 substances [274] and various chemicals in tobacco smoke have been identified as having high toxicity to respiratory cilia [275–277]. Additionally, tobacco smoke exposure has been shown to increase pro-inflammatory cytokines such as TNF-α contributing to harm respiratory mucosa [278, 279]. There is clear evidence in the literature that tobacco smoke, either through active smoking or passive exposure to secondhand smoke, contributes to CRS [280]. The prevalence of CRS has been reported to be higher in smokers [281, 282]. The impact of tobacco smoke exposure on endoscopic sinus surgery (ESS) clinical outcomes has been investigated and smokers have a less favorable response to ESS [283, 284]. It has been reported that both secondhand smoke-sensitive and –nonsensitive individuals had increased symptoms of rhinorrhea, nasal congestion, and headache following sidestream smoke exposure [285]. Individuals with known tobacco smoke sensitivity had more severe symptoms than those without previous history of secondhand smoke-related rhinitis. This study suggests that certain people may be predisposed to the effects of tobacco smoke in terms of increased sinonasal physiological and symptom response. Patients with sensitivity to tobacco smoke may be at higher risk for developing chronic upper respiratory inflammation and disease, such as CRS in response to secondhand smoke [280].

Studies have examined the pathophysiologic effects of tobacco smoke on sinonasal mucosa in an effort to explain the biologic rationale for the clinical association of smoke and CRS. The deleterious effects of cigarette smoke relevant to CRS may include alterations in secretion and ciliary beat frequency [286] as well as the induction of bacterial biofilms [287]. Tobacco smoke has also been demonstrated to have adverse effects on olfactory mucosal metaplasia [288] and may adversely impact olfaction [289, 290]. Based on *in vitro* data, it has been proposed that tobacco smoke in combination with viral infection contributes to acute exacerbations and eosinophilic inflammation in CRS patients [291]. Reactive oxygen and reactive nitrogen species from tobacco smoke induces pro-inflammatory cytokine secretion [287], epithelial apoptosis [292, 293] and diminished airway epithelial barrier function [294]. The innate immune function of sinonasal epithelium has become a significant area of research as a potential cause of CRS. These studies have shown alterations in locally expressed pattern receptors such as Toll-like receptors (TLRs) and innate immune effector proteins such as β-defensins and complement components in CRS sinonasal epithelium [295, 296]. It has been reported that tobacco smoke has immunosuppressive effects by suppressing monocyte-derived macrophage function as well as by inhibiting inflammatory cytokines through the suppression of the TLR-mediated pathway in human bronchial epithelial cells [297].

The suggestion we get from the overall data is that tobacco smoke is a likely contributor of inflammation in CRS in exposed individuals, however there is little evidence of the role of tobacco smoke as a causal agent of CRS. In particular, it was suggested in a recent study that in contrast to the lower airway, the upper airway did not appear to be as affected by the pro-inflammatory effects of tobacco smoke over time [298]. Outcome studies have also failed to show a strong negative effect from smoking [299]. So far, there is little evidence to show that tobacco smoke is a cause of CRS.

Epidemiologic and pathophysiologic studies demonstrate that lower and upper airway diseases often coexist. Chronic obstructive pulmonary disease (COPD) is a respiratory disease, but also is a systemic disorder that may be accompanied by weight loss, muscle weakness, decreased functional capacity, anemia and osteoporosis. The severity of the disease is defined by spirometric measurements [300]. Tobacco smoke is the most important risk factor for COPD, and as mentioned above, has a devastating potential for the nasal mucosa, and may cause nasal symptoms [301], impairment of mucociliary function [302], pathological signs of mucosal inflammation [303], and other changes [304]. Patients with COPD remain less studied than those with asthma in spite of the fact that a majority of COPD patients present at an academic unit of respiratory disease experience sinonasal symptoms [305, 306]. The frequency of sinonasal symptoms such as nasal discharge, nasal obstruction, and sneezing in COPD has been reported to be as high as 75 – 88% [305, 307]. Recently, a high number of patients with bronchiectasis have shown to be present with rhinosinusitis symptoms, radiologic abnormalities on CT scans [308] and have a reduced smell capacity [309]. Also, it has been shown that COPD and CRS frequently coexist [310]. Several pro-inflammatory mediators have been found in nasal lavages of COPD patients [306] and nasal symptoms corresponded with the overall impairment of the quality of life [305]. Activation of nasal neutrophils and capacity of producing secretory response to histamine were found to be increased in cases with COPD and nasal complains, compared to cases with COPD alone. Nasal IL-8 was shown to be higher in COPD patients and this increase was correlated with the increase in IL-8 level and bacterial load in sputum [303, 311–314]. The symptoms of CRS might be attributed to the systemic reflection of the inflammatory process in COPD. The connection between COPD and CRS could result from the simultaneous irritation of the lower and upper airways by exhaling tobacco smoke through the nose. Such an association could be demonstrated by the investigation of inflammatory cells and markers and the examination of tissue samples throughout the affected portions of the respiratory mucosa [310]. Further study is needed to disclose the immunopathologic mechanisms resulting in the co-occurrence of COPD and CRS.

## Pharmacotherapy

### Therapeutic modalities

#### Intranasal corticosteroids

Intranasal corticosteroids (INS) are helpful in all types of CRS. Their efficacy is supported by a high level of evidence (1a recommendation) and, therefore, they are the cornerstone of maintenance treatment [5]. Available data support the use of beclomethasone dipropionate, budesonide, flunisolide, fluticasone propionate, mometasone furoate and tixocortol pivalate [244], although mostly outside of the indication. Furthermore, the delivery system appears to matter. Nasal drops and irrigation with appropriate post application positioning appears to add clinical benefit [315]. Long-term use of nasal drops and irrigation has not been adequately studied and systemic adverse effects, including increases of intraocular pressure, require monitoring [10].

#### Systemic corticosteroids

Randomized double-blind, placebo controlled trials were performed with prednisolone 50 mg daily for 14 days [316] and methylprednisolone for 20 days [92] as a treatment for CRSwNP. Improvements were seen in rhinosinusitis outcome measure scores, extent of the disease on magnetic resonance imaging scanning, reduction in polyp size, and levels of eosinophil cationic protein (ECP), interleukin 5 and IgE in nasal secretions.

#### Systemic antibiotics

Antibiotics are acknowledged as valuable for acute exacerbations of CRS [3, 2]. According to the Infectious Diseases Society of America, amoxicillin-clavulanate is recommended as empiric antimicrobial therapy for acute bacterial rhinosinusitis in both adults and children [317]. However, in the absence of acute exacerbation, the use of antibiotics for CRS is controversial because of lack of evidence from well controlled clinical trials. The most appropriate patients with CRS for antibiotic treatment are those with persistent purulent drainage and documented infection with pathogenic organisms such as *Staphylococcus aureus*, *Pseudomonas aeruginosa* or other pathogens. These organisms can be associated with either acute or chronic infection. Eradication of infection also requires consideration of whether sinus aeration and adequate mucociliary clearance can be restored.

Long-term systemic macrolide antibiotic treatment has been advocated primarily as a treatment for CRSsNP [5]. In a study graded as level Ib evidence, in contrast to the placebo group, patients in the roxithromycin group showed a statistically significant change from baseline at 12 weeks in the SNOT-20 score, saccharine transit time and nasal endoscopy findings [260]. Better responses were seen in the subset of patients with a normal serum IgE level [260]. In a study by Videler et al, 60 patients with CRSsNP or CRSwNP were randomized to receive azithromycin versus placebo 500 mg daily x 3 days, then 500 mg weekly for 11 weeks [318]. Multiple clinical assessments were used, including symptom scoring, quality of life assessment, rigid nasal endoscopy, peak nasal inspiratory flow and endoscopically-guided middle meatus cultures. No significant differences were found between groups at the end of treatment. It is possible that inclusion of patients with elevated IgE levels or CRSwNP may have contributed to the negative results of this study.

Regarding CRSwNP, doxycycline given over 20 days also demonstrated efficacy compared to placebo in reducing nasal polyp size [92].

#### Topical antibiotics

There is some evidence of efficacy for nasal irrigations or nebulizations of antibiotics for CRS [319]. The highest level of evidence derives from prospective observational studies of post-surgical patients employing culture-directed therapy [320]. Most studies involved nebulized antibiotics for 3-6 weeks. Endoscopic improvement and an increase in infection-free interval were reported. In contrast, published placebo controlled trials failed to show benefit but were quite limited in scope and numbers of patients.

#### Intranasal and systemic antifungals

Neither topical antifungal treatment (sprays and irrigations) nor systemic terbinafine have been established as beneficial for treatment of CRS. A double-blind, placebo-controlled trial of topical amphotericin B involving 24 patients treated for 6 months produced a small but statistically significant improvement in sinus mucosal thickening [231] without improvement in symptoms. However, a subsequent double blind, placebo-controlled trial of 116 patients treated for 3 months failed to show efficacy over placebo [234]. A 12-week randomized controlled clinical trial of oral terbinafine 625 mg daily versus placebo also failed to show efficacy in terms of symptomatic or radiographic improvement for the treatment of CRS in 56 patients [321].

#### Antileukotrienes

These agents have been advocated as adjuncts to INS for the treatment of CRSwNP. Modest benefit has been noted after 1-3 months of montelukast or the 5-lipoxygenase inhibitor zileuton in studies lacking placebo control [322, 323]. However, placebo-controlled studies have mostly failed to demonstrate benefit of montelukast for nasal polyposis, and zileuton has not been subjected to a placebo-controlled trial [5].

### Adjunctive therapies

A Cochrane review of 8 studies using various forms of saline sprays and irrigation performed 1-4 times daily found that intranasal saline is an effective adjunctive treatment for CRS [324]. Saline irrigation provides a subjective sense of freshening, rinses away allergens and irritants, removes secretions, improves mucociliary clearance, and reduces postnasal drainage. An isotonic concentration is generally preferred to hypertonic saline. Intranasal lavage (with at least 200 ml of warmed saline per side) can be performed with over-the-counter devices such as squeeze bottles, syringes and pots. Appropriate cleaning is required to avoid contamination of the device.

The evidence does not currently support the use of mucolytics, oral decongestants, or protracted administration of intranasal decongestants for CRS. However, therapies of associated conditions may aid the management of CRS. These include antihistamines, environmental control to reduce problematic exposures and allergen immunotherapy for patients with allergic rhinitis, and H2 antagonists and proton pump inhibitors for patients with laryngopharyngeal reflux. For patients with aspirin-exacerbated respiratory disease (AERD), aspirin desensitization followed by daily aspirin therapy has been reported as beneficial for control of nasal polyps, although placebo-controlled trials have not been conducted [325–329].

### CRS Pharmacotherapy

There is a relative paucity of controlled studies for this indication. The design and interpretation of CRS clinical trials has been hindered by the heterogeneity of the disease, a deficiency of uniform definitions for disease subtypes, incomplete understanding of the underlying pathologies, and a lack of useful and standardized clinical and laboratory endpoints to measure response to therapy. The most comprehensive treatment recommendations for CRS are put forth in the EPOS consensus document [5]. Recommendations are categorized into 3 major subtypes: CRSsNP, CRSwNP and AFRS. Recommendations are also stratified according to disease severity, using a visual analogue scale (VAS) of 0 (none) to 10 (most severe) [244].

**CRSsNP** (with evidence level)

1. Initially intranasal saline (A/Ib) and INS (A/Ib)
2. If after 3 months not improved, perform culture, institute long-term macrolide therapy (A/Ib)
3. If improved, continue intranasal saline and INS with/without macrolide therapy
4. If after the 3 months lack of response to this strategy, consider CT scanning, surgery

Alternative recommendations to consider for initial treatment: 10 day course of oral corticosteroids plus 3-4 week course of oral antibiotics (empirically selected or guided by culture). Amoxicillin-clavulanate is an excellent choice for most patients. Clindamycin or moxifloxacin are useful for patients with penicillin allergy [10]. Macrolide therapy may also be considered for initial treatment in those with moderate/severe symptoms.

**CRSwNP** (with evidence level)

1. Initially INS (A/Ia) in double dosage, may be tapered down if disease under control
2. If after 3 months not controlled, switch to INS drops [1) (A/Ib), review after 3 months
3. Because of frequent mucosal colonization with *S aureus*, doxycycline, 200 mg day 1, then 100 mg for 20 days [92], initiate short course of oral steroids (e.g., prednisolone 50 mg X14 days) [316] (A/Ia)
4. If after 3 months not controlled, consider CT scanning, surgery

Alternative recommendations to consider for initial treatment:

1. Initially INS drops(A/Ib), a short course of oral steroids (A/Ia) and doxycycline100mg/day for 3 weeks [92]

Additional recommendations to consider for maintenance treatment: treatment of underlying allergic rhinitis, aspirin desensitization followed by daily aspirin therapy for post-surgical management of patients with AERD, and antileukotriene agents [10].

**AFRS** (with evidence level)

1. Remove fungal mass and polyps (this usually requires endoscopic sinus surgery)
2. Systemic steroids post-operatively (usually prednisolone started at 0.5 mg/kg daily with tapering over a few weeks or longer, depending on control of symptoms and mucosal disease) [10]. (Ia/A)
3. INS saline, INS and INS drops can be considered for maintenance treatment as in CRSwNP [10]. (IV/D)
4. Intranasal or systemic antifungal agents have no proven efficacy.

Additional recommendation to consider for initial treatment: preoperative systemic corticosteroids may help to improve sinus landmarks for surgery [10].

## Surgical interventions

It is generally accepted that surgical intervention should be considered when symptomatic chronic rhinosinusitis (CRS) is refractory to appropriate medical therapy indicating that the sinus mucosal inflammation is not adequately controlled [5]. The outcome (efficacy and safety) of surgery at the individual level is influenced by two broad categories of factors; patient-related factors such as the phenotype of CRS, smoking or occupational exposure, compliance to medication; and surgeon-related factors such as the surgeon’s skills, the surgical techniques employed, and postoperative management. While endoscopic sinus surgery (ESS) is widely considered as the standard surgical intervention for CRS, the optimal techniques for surgical treatment of CRS without nasal polyps (CRSsNP) or CRS with nasal polyps (CRSwNP) are still under debate.

Major advances in nasal endoscopy [330] and computed tomography (CT) [331] over the last three decades have resulted in the progress from sinus surgery preferentially involving external approaches using a headlight to surgery involving endoscopic intranasal approaches, namely ESS [332]. Furthermore, advances in instrumentation, such as through-cutting instrumentation, angled suction irrigation drills, powered microdebriders, high-quality three-chip or digital cameras, and interactive computer-assisted frameless stereotactic surgical navigation systems have enabled the surgeon to perform precise and rapid dissections with mucosal preservation under enhanced visualization [333].

Based on work of Messerklinger in 1978 [330], it is now recognized that obstruction of the ostiomeatal complex (OMC) is the critical etiologic factor in the pathogenesis of CRSsNP and that mucosal damage was reversible [334]. The abbreviation FESS was first coined in 1985 by Kennedy and colleagues [335], aimed at reversing the mucosal inflammation and the mucociliary dysfunction of sinuses by removing the diseased tissue from the target drainage area of the OMC. However, “*…simply draining involved cells or sinuses may be insufficient in chronic disease*” [336, 337]. Specifically in CRSwNP, persistent inflammation is likely to determine the long term outcome, and anti-inflammatory strategies are mandatory.

Although it has been reported that the obstruction of the sinus ostia initiates a cascade leading to rhinosinusitis [338], the likelihood that improvements in sinus ventilation alone are sufficient to cure the mucosal inflammation, especially in CRSwNP, is counterintuitive. Surgical treatment should be considered as an adjunct to the medical treatment of CRS rather than a stand-alone procedure [339], at least for most of the patients, if not for all. While a recent systemic review from the Cochrane database indicated that the surgical procedure did not confer an additional benefit to the treatment of CRSsNP [340]; a more recent comparative multi-centre study with 1-year follow-up has demonstrated that ESS treatment led to significantly greater QOL improvements than medical treatment in patients with CRSsNP or CRSwNP who had previously failed to improve with medical treatment [341].

In general, ESS is effective and safe for patients with CRS resistant to medical treatment [342]. Dalziel and colleagues [343] reviewed a total of 42 randomized controlled trials, nonrandomized comparative studies, and case series describing outcomes associated with FESS for the excision of nasal polyps, and reported that FESS led to symptomatic improvement in up to 98% of CRSwNP patients with low frequency of major complications (from 0 to 1.5%). Similarly, a systematic review of studies that investigated symptom severity scores to analyse at least three major CRS criteria in adults recently demonstrated that ESS led to symptomatic improvements in both CRSsNP and CRSwNP [344]. A large prospective study investigating long-term outcomes in a cohort of 1459 patients who had undergone surgery for CRS (with or without nasal polyps) has recently demonstrated that although sinonasal surgery was both safe and effective in reducing the symptoms associated with CRS over a 5-year period, the overall revision surgery rate over 5 years was as high as 19.1%; with the revision surgery rate for CRSwNP patients being higher than that for CRSsNP patients (21% vs. 16%) [345]. It is noteworthy that differences in the classification of CRS (CRSsNP or CRSwNP), the instrument of evaluation (subjective or objective), and the length of follow-up are all likely to contribute to wide variations in the efficacy and safety of ESS.

The extent of ESS and the techniques involved should be tailored to the classification, phenotype, and severity of the disease. Although we have not reached a consensus on how to surgically resolve CRSw/sNP, the techniques and technologies deployed in the surgical treatment of CRS continue to evolve along with our understanding of the pathophysiology of chronic sinus inflammation.

## Treatment options (adult, children): biological approaches

Two main innovative biological therapies have being investigated for efficacy and safety in adult patients with chronic rhinosinusitis with nasal polyposis (CRSwNP): omalizumab, a recombinant humanized monoclonal antibody against free immunoglobulin E (IgE), and reslizumab and mepalizumab, two humanized antibodies against interleukin (IL)-5.

### Humanized monoclonal antibody against IgE

Omalizumab is approved for the treatment of severe allergic asthma, otherwise failing to respond to asthma treatment, with IgE serum levels between 30 and 1500 kU/L. It has been demonstrated that total IgE levels are increased in nasal secretions, polyp tissue, and serum of patients with CRSwNP, comparable to findings in asthma [195]. In fact, it is now clear that there is true local IgE formation in nasal polyp tissue, which might be further triggered by staphylococcal superantigens [169]. Two studies have published case report series in patients being treated with omalizumab for their comorbid severe asthma: in a pilot study 4 treated patients showed a reduction in polyp size but not in CT scores [346], and in a series of 19 (13 postoperative) treated patients [347], all showed a reduction of polyp size, use of intranasal corticosteroids, and further sinonasal surgery. However, a negative underpowered study also including CRSsNP patients has also been published [348]. A recent randomized, double-blind, placebo-controlled study with 24 patients with CRSwNP and co-morbid asthma treated with omalizumab [196] showed a significant reduction of polyp size, an improvement of bronchial and nasal symptoms, including smell, quality of life, and sinus CT scan. The study suggests that omalizumab does work in allergic and non/allergic subjects. Patients with CRSwNP and comorbid asthma, preferentially after sinonasal surgery, may obtain a clear clinical benefit from omalizumab therapy in both the lower and upper airways.

### Humanized antibodies against IL-5

IL-5 is one of the most important eosinophil activating factors orchestrating airway eosinophilic inflammation [177]. The levels of IL-5 are elevated in nasal secretions, polyp tissue, and serum of Caucasian patients with CRSwNP. Two randomized, double blind, placebo-controlled trials using anti-IL-5 antibodies, reslizumab [349] and mepolizumab [350], have shown to reduce the number of blood and tissue eosinophils and the size of nasal polyps with a greater benefit for patients with high levels of IL-5 in nasal secretions. Based on these studies, the 2012 update of EPOS consensus [6] recommended (grade A) the use of humanized antibodies against IL-5 for the treatment of patients with CRSwNP [351].

## Unmet needs in chronic rhinosinusitis

Although it recently has been established that chronic rhinosinusitis is a frequent disease in Europe and the US, data from other continents are scarce, but are needed to recognize differences and factors associated with CRS prevalence. The tools to screen for CRS in epidemiological studies need to be further developed, specifically in terms of differentiation between CRS and other chronic upper airway diseases such as allergic rhinitis. Factors predisposing to CRS such as smoking need to be confirmed and others identified, especially in childhood and adolescence.

**Figure 3:**
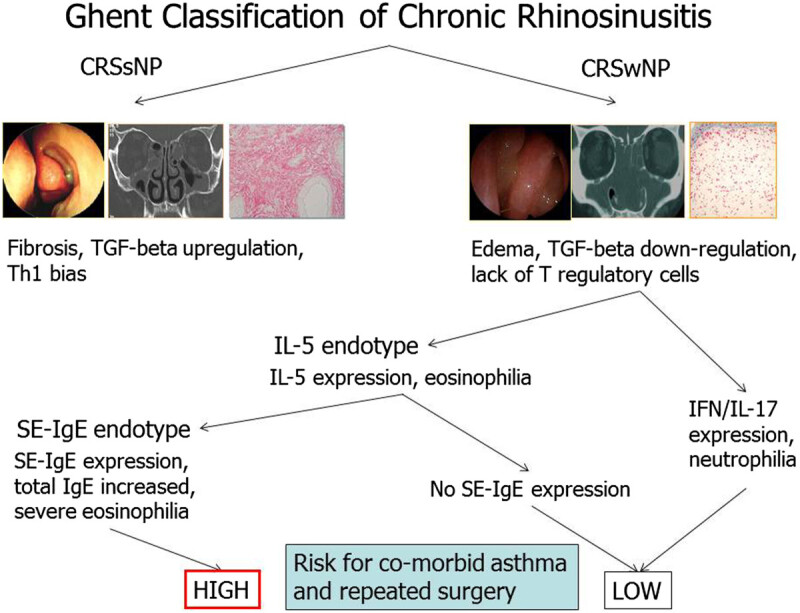
**"Pheno- and endotype" CRS.** Pheno- and endotyping of CRS based on the recently published findings on asthma comorbidity and recurrence after surgery (Ghent classification of CRS).

We realize that CRS is an expensive disease with high burden to the patient and the society; however, tools to define disease severity and identify factors impacting on pharmaco-economics are not well defined yet. The influence of disease subtype and patterns of inflammation and co-morbidities on burden and costs need to be defined, and treatment approaches need to be evaluated in terms of appropriateness.It has been established that co-morbid asthma is frequent in CRS patients, however, the factors linking these diseases specifically in late-onset non-atopic asthma are only partially understood, and a better understanding of the subgroups of CRS patients likely to develop asthma [and disease recurrence) is necessary. This goes in parallel with the need to “pheno- and endotype” CRS, most likely involving tissue biomarkers and their surrogates in serum and secretions (Figure 3). Again, these studies need to be performed in various areas of the world, as inflammation in CRS may considerably vary from region to region, and also may show alterations over time.

Hypothesis-free approaches, based on –omics [genomics, epigenomics, proteomics, metabolomics etc.), will ideally lead to meaningful clusters of disease and offer new etiological and pathophysiological pathways. However, this will ask for a profound validation of those approaches for upper airway disease, and a successful translation of specific findings into clinical medicine. Once identified, those clusters or endotypes will form the basis for further research into genetics, environmental factors and microbiota, as they allow splitting up the umbrella term of chronic rhinosinusitis.

At the same time, the natural course of disease and disease endotypes is far from clear, and factors impacting on it, as the environment and pollution, the airway microbiome and exposure to antibiotics, and nutritional behavior, need to be understood. We need to unravel the interactions between the microbiota present in different forms and locations in the sinuses [planktonic, biofilms, intramucosal and intracellular manifestations) and the type of inflammation, building up on observations on the impact of Staphylococcus aureus on the severity of CRS disease. The interaction of the invaders with the innate and adaptive immune system awaits clarification, from the immune proteome of specific bacteria to the PAMPs and DAMPs of the airway mucosa. Also, prior or due to the mucosal inflammation, specific gaps in the mucosal defense are likely to play an important role, including the immunity against bacteria and viruses, and possibly also fungi.

With the endotyping of CRS, new diagnostic tools and therapeutic interventions in carefully selected patients will be possible, but need evaluation and positioning within the management algorithm. Diagnosis of CRS will not only rely on nasal endoscopy and CT scanning, but include biomarkers. Furthermore, the current standards including sinus surgery will be challenged, and most probably changed especially in severely diseased patients with co-morbidity and disease recurrence. These changes offer new perspectives for patients currently inadequately managed, but also challenges to our daily practice.

## Authors’ information

Claus Bachert and Ruby Pawankar are first authors.

## Authors’ original submitted files for images

Below are the links to the authors’ original submitted files for images. Authors’ original file for figure 1 Authors’ original file for figure 2 Authors’ original file for figure 3
