# Supplementary figures and images for: ICON: chronic rhinosinusitis
Source: World Allergy Organ J. 2014 Oct 27;7(1):25. doi: 10.1186/1939-4551-7-25 (PMC4213581; doi:10.1186/1939-4551-7-25)

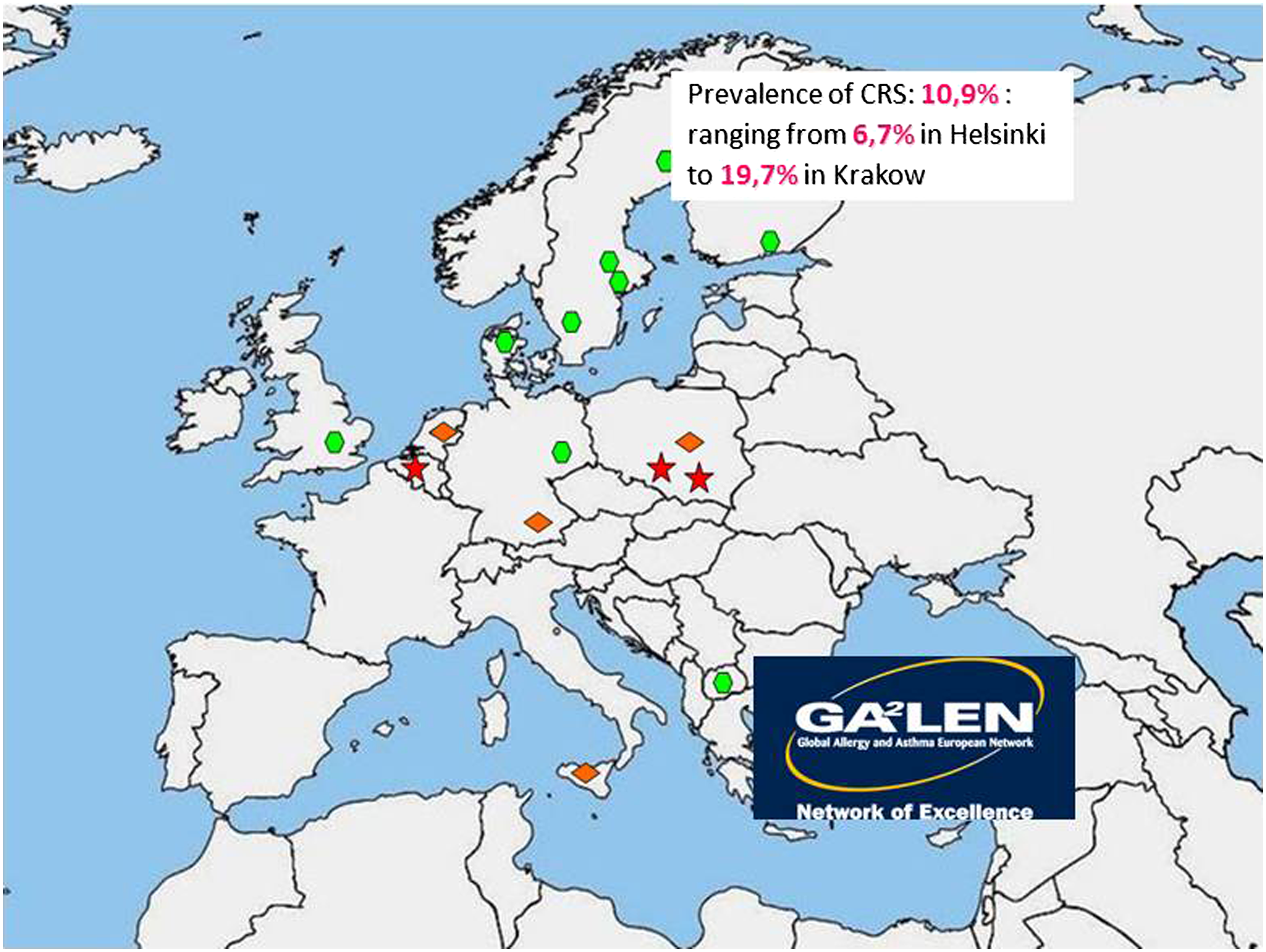

Supplement: Supplementary file 1 — Authors’ original file for figure 1 [file 40413_2014_69_MOESM1_ESM.tiff]

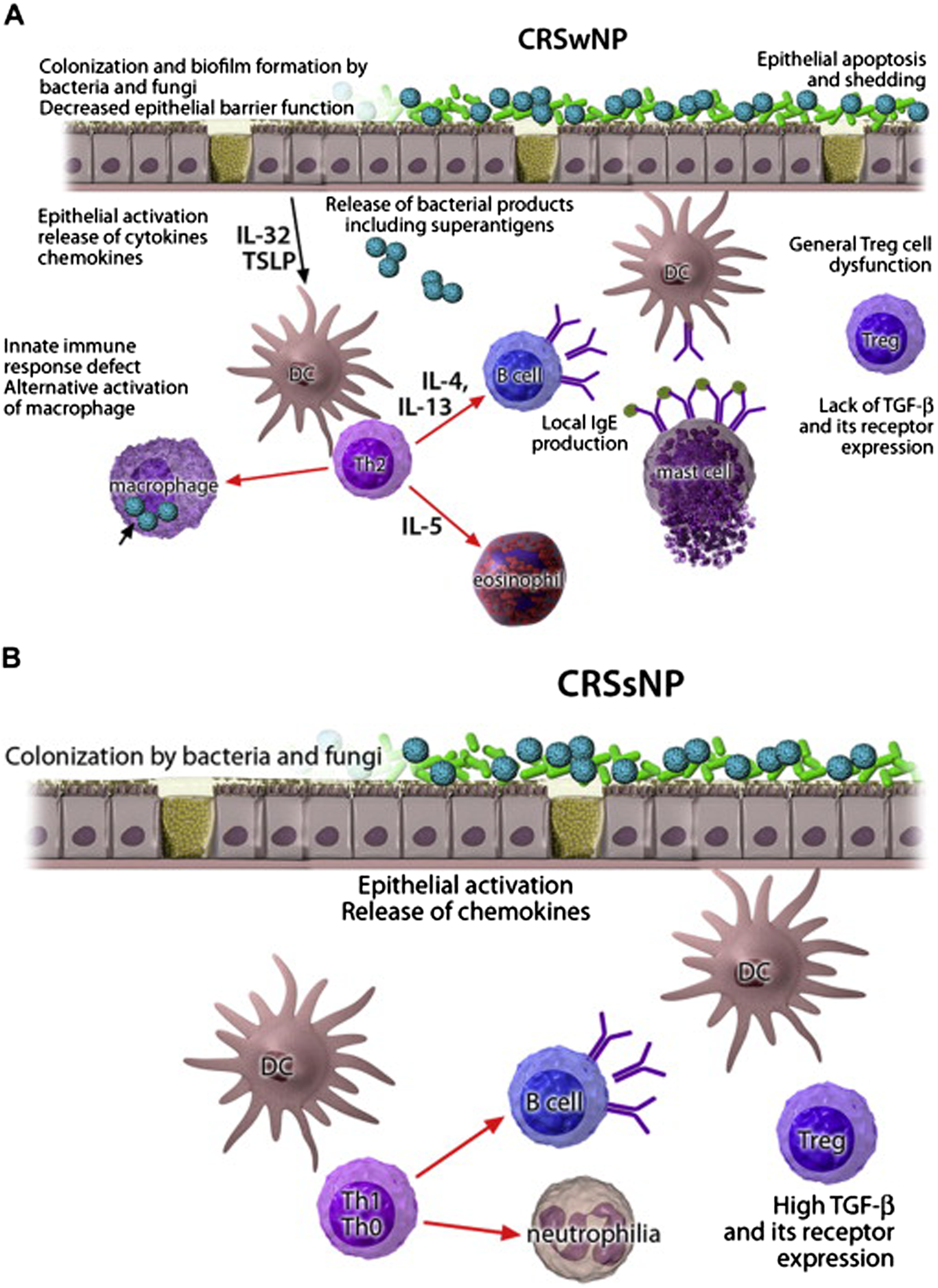

Supplement: Supplementary file 2 — Authors’ original file for figure 2 [file 40413_2014_69_MOESM2_ESM.tiff]

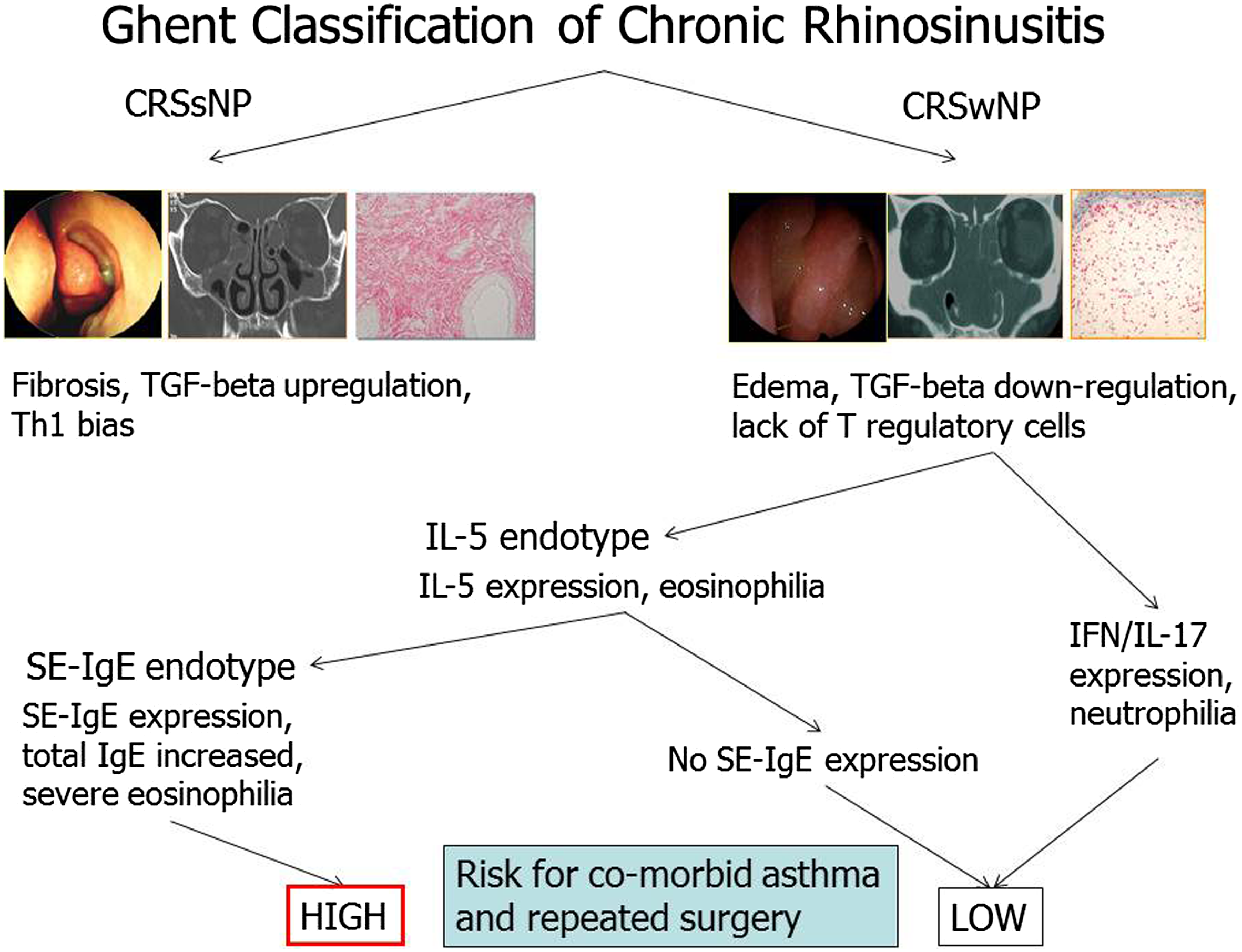

Supplement: Supplementary file 3 — Authors’ original file for figure 3 [file 40413_2014_69_MOESM3_ESM.tiff]
